# Scanning Electrochemical Microscopy Imaging during Respiratory Burst in Human Cell

**DOI:** 10.3389/fphys.2016.00025

**Published:** 2016-02-05

**Authors:** Hiroyuki Kikuchi, Ankush Prasad, Ryo Matsuoka, Shigeo Aoyagi, Tomokazu Matsue, Shigenobu Kasai

**Affiliations:** ^1^Graduate Department of Environmental Information Engineering, Tohoku Institute of TechnologySendai, Japan; ^2^Biomedical Engineering Research Center, Tohoku Institute of TechnologySendai, Japan; ^3^Hokuto Denko CorporationAtsugi, Japan; ^4^Graduate School of Environmental Studies, School of Engineering, Advanced Institute for Materials Research, Tohoku UniversitySendai, Japan

**Keywords:** biosensors, THP-1 cells, respiratory burst, SECM imaging, hydrogen peroxide

## Abstract

Phagocytic cells, such as neutrophils and monocytes, consume oxygen and generate reactive oxygen species (ROS) in response to external stimuli. Among the various ROS, the superoxide anion radical is known to be primarily produced by nicotinamide adenine dinucleotide phosphate hydrogen (NADPH) oxidase. In the current study, we attempt to evaluate the respiratory burst by monitoring the rapid consumption of oxygen by using scanning electrochemical microscopy (SECM) imaging. The respiratory burst was measured in a human monocytic cell line (THP-1 cells) derived from an acute monocytic leukemia patient under the effect of the exogenous addition of phorbol 12-myristate 13-acetate, which acts as a differentiation inducer. SECM imaging composed of a microelectrode was used to compare oxygen consumption between normal cellular respiration and during respiratory burst in THP-1 cells. Two-dimensional respiratory activity imaging was performed using XY-scan. In addition, the quantitative evaluation of oxygen consumption in THP-1 cells was performed using a Z-scan. The results obtained show higher consumption of oxygen in cells undergoing respiratory burst. SECM imaging is thus claimed to be a highly sensitive and appropriate technique compared to other existing techniques available for evaluating oxidative stress in human cells, making it potentially useful for widespread applications in biomedical research and clinical trials.

## Introduction

Living organisms bear defense mechanisms in which immune cells, such as neutrophils and monocytes, play pivotal roles in responding to and killing foreign bodies that invade the living system (Forman and Torres, 2002; Halliwell and Gutteridge, 2007). Phagocytic cells, such as neutrophils and monocytes, produce reactive oxygen species (ROS) during phagocytosis (Forman and Torres, 2002). Abrupt glucose and oxygen consumption, termed respiratory burst, is known to occur and is associated with the formation of several oxygen containing compounds via the activation of nicotinamide adenine dinucleotide phosphate hydrogen (NADPH) oxidase (Forman and Torres, 2002). NADPH oxidase leads to the formation of superoxide anion radical (O_2_^•−^) in the cell, which further dismutate, leading to the formation of hydrogen peroxide (H_2_O_2_) (Halliwell and Gutteridge, 2007). The formation of H_2_O_2_ then acts as a precursor for the generation of more toxic oxygen compounds, such as hydroxyl radical (HO^•^), etc. (Auchere and Rusnak, 2002). Myeloperoxidase, which is most abundantly expressed in neutrophil granulocytes, utilizes H_2_O_2_ and halide ions (typically Cl^−^) and leads to the generation of hypochlorite, which is highly toxic (Harrison and Schultz, 1976; Robinson, 2008). In our previous study, we demonstrated the enhancement of reduction current for H_2_O_2_ during the differentiation process of leukocytes and THP-1 cells using microelectrode (Shigenobu et al., 2005; Inoue et al., 2010). The respiratory burst in THP-1 cells was induced by exogenous addition of phorbol 12-myristate 13-acetate (PMA) (Shigenobu et al., 2005). The respiratory burst under PMA addition is known to occur via activation of protein kinase C (PKC), which then leads to activation of NADPH oxidase (Castagna et al., 1982; Kikkawa et al., 1983). The THP-1 cells were differentiated to macrophages using 20 nM PMA as the stimulant. In the current study, the kinetics of the simultaneous real-time measurement of oxygen consumption and generation of H_2_O_2_ using electrochemical biosensors is demonstrated to establish a correlation. The main goal of the current study is to introduce scanning electrochemical microscopy (SECM) imaging of oxygen consumption in immune cells for the first time, which is associated with ROS generation during respiratory burst. Using SECM, we measured the change in the value of the oxygen reduction current during cellular respiration and respiratory burst by moving a microelectrode back and forth around the vicinity of the cells. The quantitative evaluation of respiratory activity of THP-1 cells was performed by Z-scan, and respiratory activity imaging of THP-1 cells was performed by XY-scan.

## Materials and methods

### Cell culture and reagents

Human monocytic leukemia cell line, THP-1 cells, was purchased from JCRB (Japanese Collection of Research Bioresources) (Cosmo Bio. Co. Ltd., Tokyo, Japan) cell bank. The cells were maintained in RPMI 1640 media supplemented with 2 mM L-glutamine and incubated at 37°C in 5% CO_2_ in a humidified atmosphere. Glucose, L-glutamine and PMA of analytical grade were purchased from Wako Pure Chemical Industries, Ltd. (Osaka, Japan), and RPMI 1640 medium was purchased from Sigma Chemical Co. (St. Louis, MO, USA).

### Equipment and methods for electrochemical measurements

Simultaneous measurements of oxygen reduction current and reduction current for H_2_O_2_ were performed using a potentiostat (HA1010mM4S; Hokuto denko Co., Ltd., Japan). Two-dimensional imaging and quantitative evaluation of respiratory activity using a 3D -cell chip were performed using SECM. For microelectrode scanning, a motor-driven XYZ-stage (Suruga Seiki, K701-20M) was located on the microscopic stage.

### Carbon and osmium-horseradish peroxidase (Os-HRP) modified carbon microelectrode

For simultaneous measurement of oxygen consumption and H_2_O_2_generation, carbon electrodes (φ = 1 mm) and Os-HRP modified carbon electrodes (φ = 1 mm) were used as working electrodes I and II, respectively. The carbon electrodes were purchased from BAS Inc., ALS Co., Ltd., Japan. The Os-HRP modified carbon electrode was prepared as follows: prior to each measurement, the carbon electrode was cleaned using the PK-3 Electrode Polishing Kit (BAS Inc., ALS Co., Ltd., Japan), followed by immobilization of a 0.5 μL aliquot of Os-HRP polymer solution (Bioanalytical System, USA) and overnight incubation at 4°C under dark conditions, allowing for the formation of a circular film on the carbon electrode. An Ag/AgCl electrode was used as a reference electrode.

### Experimental conditions for simultaneous measurement of oxygen reduction current and reduction current for H_2_O_2_

THP-1 cells were measured in a 6 well Repro plate (IFP, Research unit for the functional peptides, Yamagata, Japan) at a density of 2.0 × 10^6^ cells/well. Cells were suspended in phosphate buffered saline (PBS) (Sigma Chemical Co., St. Louis, MO, USA) in the presence of 11.4 mM glucose. PMA was added dropwise to a final concentration of 20 nM and simultaneous reduction currents were measured. The oxygen reduction current was measured at −0.5 V vs. Ag/AgCl and the reduction current for H_2_O_2_ was measured at 0.0 V vs. Ag/AgCl at room temperature. Chronoamperometric response of standard H_2_O_2_ solution in real-time was also measured in the concentration range of 0.1–0.3 nM. Subsequent reduction current for H_2_O_2_ was monitored using Os-HRP modified carbon electrode (φ = 1 mm) with Ag/AgCl as reference electrode (Supplementary Data 1).

### 3D-cell chip preparation

Silicon substrates were fabricated by anisotropic etching. Silicon wafers with dimensions of 2.5 cm × 1 cm × 1 mm and 2.5 cm × 2.5 cm × 230 μm were prepared. The pyramid-like cavities were etched into the silicon wafers in two different dimensions. The sizes of the larger and smaller openings for qualitative estimation (XY-scan) were 370 and 100 μm, respectively, whereas the openings for quantitative estimation (Z-scan) were 1550 and 200 μm, respectively.

For SECM imaging, a collagen-cell mixture was prepared at 4°C by mixing Type I collagen (Cellmatrix Type I-A, Nitta Gelatin), liquid culture medium, and PBS buffer in a 7:2:1 ratio. The final cell density in the 14 nL pyramid well (for XY-scan) was maintained at 2.4 × 10^2^ cells/well, whereas the final cell density in the 900 nL pyramid well was maintained at 3.0 × 10^3^ cells/well. After inserting the collagen-cell mixture into the fabricated silicon well, it was incubated at 37°C in 5% CO_2_ for 5 min for conversion into gel. For measurement of normal cellular respiration, THP-1 cells were added just prior to incubation at 37°C. During measurement of respiratory burst, PMA was added under the same experimental conditions to the THP-1 cells at a final concentration of 20 nM and measurements were recorded after ~20 min of PMA addition.

### Experimental conditions for SECM imaging

For SECM imaging, the working electrode was a Pt microelectrode (φ = 10μm), the reference electrode was an Ag/AgCl electrode, and the measurement solution was PBS buffer containing 11.4 mM glucose. SECM imaging of cellular respiration and respiratory burst was obtained by XY-scanning. The XY-direction scanning zone was 500 × 500 μm. The scanning speed was maintained at 20 μm/s with a resolution of 10 μm. The change in the value of the oxygen reduction current was measured with an applied voltage of −0.5 V vs. Ag/AgCl.

### Quantitative evaluation of respiratory activity using SECM

The oxygen reduction current was measured using a platinum (Pt) microelectrode (φ = 20 μm) at room temperature in 40 mM PBS buffer containing 11.4 mM glucose. An Ag/AgCl electrode was used as a reference electrode. With the initial position of the working electrode from the silicon substrate at 30 μm in the Z direction, the potential was held at −0.5 V vs. Ag/AgCl. This electrode was moved back and forth in the Z-direction 3 times from the vicinity of the cells up to 300 μm at a speed of 10 μm/s and the change in the value of the oxygen reduction current was measured.

## Results and discussion

### Simultaneous measurement of oxygen consumption and hydrogen peroxide production

**Figure 1:**
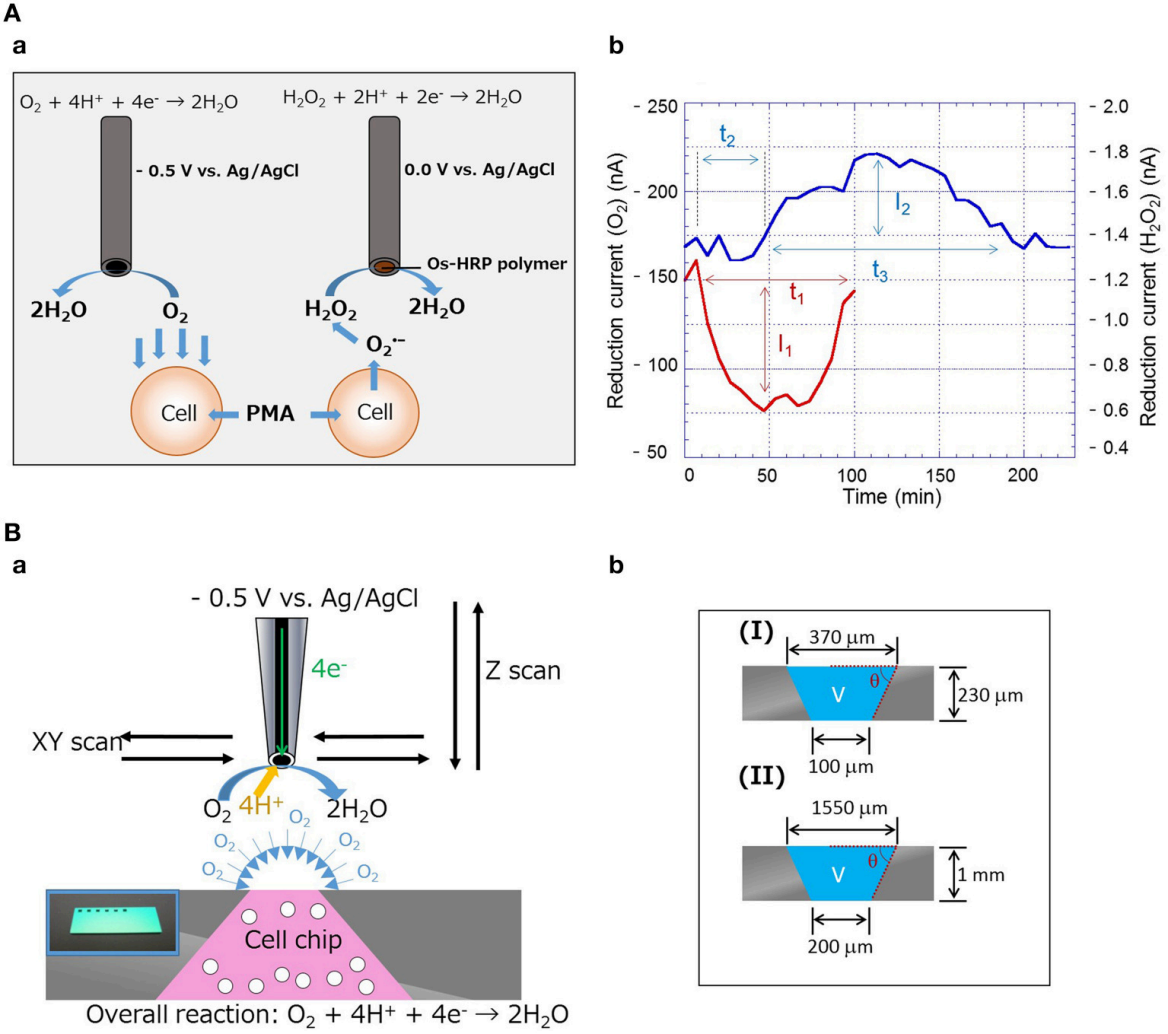
**(Aa)** Schematic diagram showing the setup for simultaneous measurement of oxygen consumption and generation of hydrogen peroxide (H_2_O_2_) using an electrochemical method. Carbon microelectrode for detection of oxygen reduction current and Os-HPR modified carbon microelectrode for detection of reduction current for H_2_O_2_. **(Ab)** Real-time monitoring of oxygen consumption and H_2_O_2_ production during respiratory burst in THP-1 cells with exogenous addition of 20 nM PMA. **(Ba)** Schematic representation of the setup for evaluation of two-dimensional (XY-scan) and quantitative respiration (Z-scan) activity in THP-1 cell chip using SECM (insert shows the photograph of the cell-chip used). **(Bb)** Diagram showing the dimensions of the cell chip prepared for XY-scan (I) and Z-scan (II).

Using the carbon and Os-HPR modified carbon microelectrode, the oxygen reduction current, and reduction current for H_2_O_2_ were measured at −0.5 V vs. Ag/AgCl and 0.0 V vs. Ag/AgCl, respectively, in the presence of PMA. A schematic representation showing the working principle of the simultaneous measurement of oxygen consumption and hydrogen peroxide generation using catalytic amperometric biosensor device is depicted in Figure 1Aa. Upon addition of PMA at a final concentration of 20 nM, changes in oxygen reduction current and reduction current for H_2_O_2_ were observed (Figure 1Ab). During the exogenous addition of PMA, the oxygen reduction current was decreased by 63 nA. The total equivalent oxygen concentration was recalculated to be ~79 μM using the standard bulk oxygen concentration as described by Hitoshi et al. (2005). The rapid decrease in oxygen reduction current was observed as a result of abrupt oxygen consumption during the respiratory burst, which continues for a span of ~80 min. On the contrary, the reduction current for H_2_O_2_ increased after approximately 20 min of PMA addition, which was observed for a period of up to 140 min, indicating that the production of H_2_O_2_ continues for a long time. From the above observations, it can be concluded that oxygen consumption and H_2_O_2_ production are dependent phenomena that are linked together.

### SECM imaging of respiratory activity using 3D-cell chip

**Figure 2:**
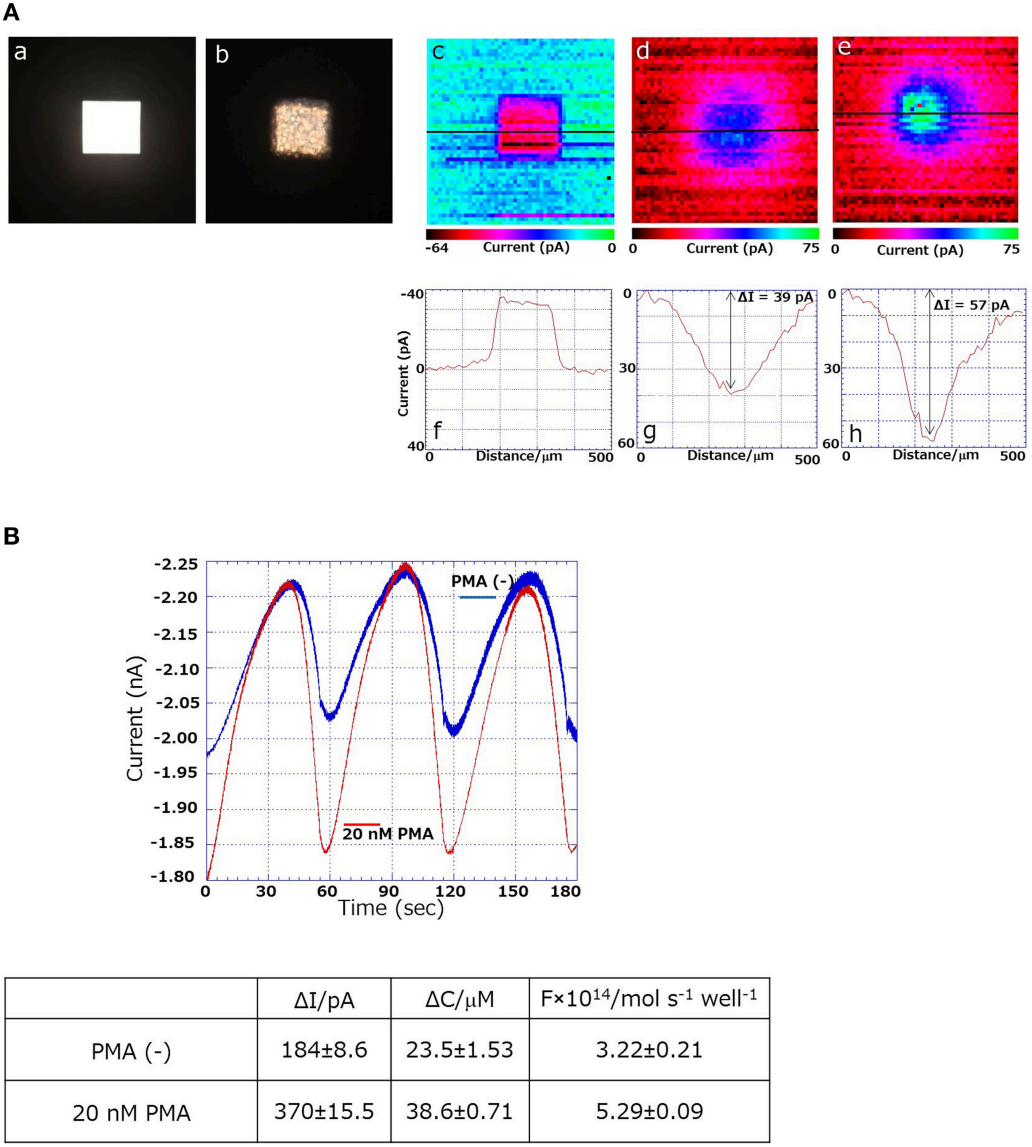
**(Aa)** Photographs of the 3D-cell chip without **(a)** and with THP-1 cells **(b)**; SECM images and changes in reduction current (in pA) using 3D THP-1 cell chip in chip containing no cells **(c,f)**; during cellular respiration **(d,g)** and respiratory burst **(e,h)**. **(B)** Comparison of magnitude of oxygen reduction current using SECM during ordinary respiration (blue trace) and during respiratory burst (red trace) (*n* = 3).

Using the 3D-cell chip, two-dimensional imaging of cellular respiration and respiratory burst was measured in THP-1 cells using SECM imaging. Figure 1Ba shows the schematic illustration on the setup and the principle of the 3D-cell chip and detection of the reduction current using SECM. The photograph of the 3D-cell chip is shown as an insert of Figure 1Ba. For the two-dimensional imaging of cellular respiration and respiratory burst, a 3D-chip of dimensions 2.5 cm × 2.5 cm × 230 μm was used with a pyramid well with the dimensions shown in Figure 1Bb. Figures 2Aa,b shows the photographs of the 3D-cell chip without and with THP-1 cells. SECM imaging was performed according to the parameters mentioned (Torisawa et al., 2006) with minor modifications as described in the material and methods section. A higher oxygen reduction current was generated in the system containing no cells, whereas the area where THP-1 cells were present showed significantly lower intensity corresponding to lower oxygen reduction current in the system containing THP-1 cells (Figures 2Ac,d). Two-dimensional SECM imaging during PMA-induced respiratory burst was also monitored (Figure 2Ae). In comparison to normal cellular respiration, the decrement in oxygen reduction current was considerably large in the case of respiratory burst. Figures 2Ac–e shows that the lower oxygen reduction current observed coincides with the location of the cell, i.e., decrease in oxygen reduction current at the center of the well (Supplementary Data 2). Oxygen reduction current is lower in the case of respiratory burst (Figure 2Ae) compared to cellular respiration (Figure 2Ad). The change in oxygen reduction current in 3D-cell chip without THP-1 cells was recorded to be -36pA (Figure 2Af). The changes in oxygen reduction current during normal cellular respiration and respiratory burst were found to be 39 and 57 pA, respectively (Figures 2Ag,h). From the topographic view of the SECM image and considering the relative intensity distribution, the diffusion layer of oxygen is observed as spherical.

### Quantitative evaluation of respiratory burst using SECM

The quantitative estimation of respiratory burst was measured using SECM by the employment of a probe microelectrode. Figure 2B shows the results using a Pt microelectrode moving back and forth in the Z-direction (*n* = 3) to determine the difference of oxygen reduction current at a distant position and in the vicinity of the cells by SECM. The oxygen reduction current was measured during cellular respiration [PMA (−), blue trace] and during the respiratory burst (20 nM PMA, red trace). The difference (ΔI) of the oxygen reduction current between the vicinity of the cells and the distant position during normal cellular respiration of THP-1 was measured as ~184 ± 8.6 pA, whereas ΔI was ~370 ± 15.5 pA during respiratory burst of THP-1. Figure 2B shows the difference in dissolved oxygen concentration between the vicinity of cells and bulk solution (ΔC) and respiration rate (F) calculated using a dissolved oxygen concentration of 209 μM (Hitoshi et al., 2005) and an oxygen diffusion coefficient of 2.18 × 10^−5^ cm^2^/s. The respiration rate during ordinary respiration was calculated to be 3.22 ± 0.21 mol/s/well, whereas the respiration rate of the respiratory burst was calculated to be 5.29 ± 0.09 mol/s/well. This result shows that during respiratory burst, the oxygen consumption was ~2 times higher than that during normal cellular respiration. In the current study, we succeeded in obtaining the difference in 2-dimensional imaging between normal cellular respiration and respiratory burst by XY-scanning. In addition, we achieved quantitative respiratory activity by sweeping the microelectrode in the Z-axis direction, which provides a precise estimation of oxygen consumption in the bulk solution. In the future, to calculate the amount of respiratory burst in cells more precisely and accurately, we plan to obtain measurements in single cells by suitably arranging electrode sizes and measurement wells. From the results presented herein, which demonstrate SECM imaging as a potent technique with very high sensitivity, SECM is projected to be a suitable technique for a wide range of applications.

## Author contributions

HK fabricated the device and performed the measurements. HK and AP analyzed and interpreted the data. AP drafted the manuscript. SK contributed to the conception and design of the work and revised it critically for important content. All authors approved the final version of the manuscript.

### Conflict of interest statement

The authors declare that the research was conducted in the absence of any commercial or financial relationships that could be construed as a potential conflict of interest.
