# Supplementary material for: Scanning Electrochemical Microscopy Imaging during Respiratory Burst in Human Cell
Source: Front Physiol. 2016 Feb 5;7:25. doi: 10.3389/fphys.2016.00025 (PMC4742556; doi:10.3389/fphys.2016.00025)
Supplement: Supplementary file 1 [file Image1.PDF]

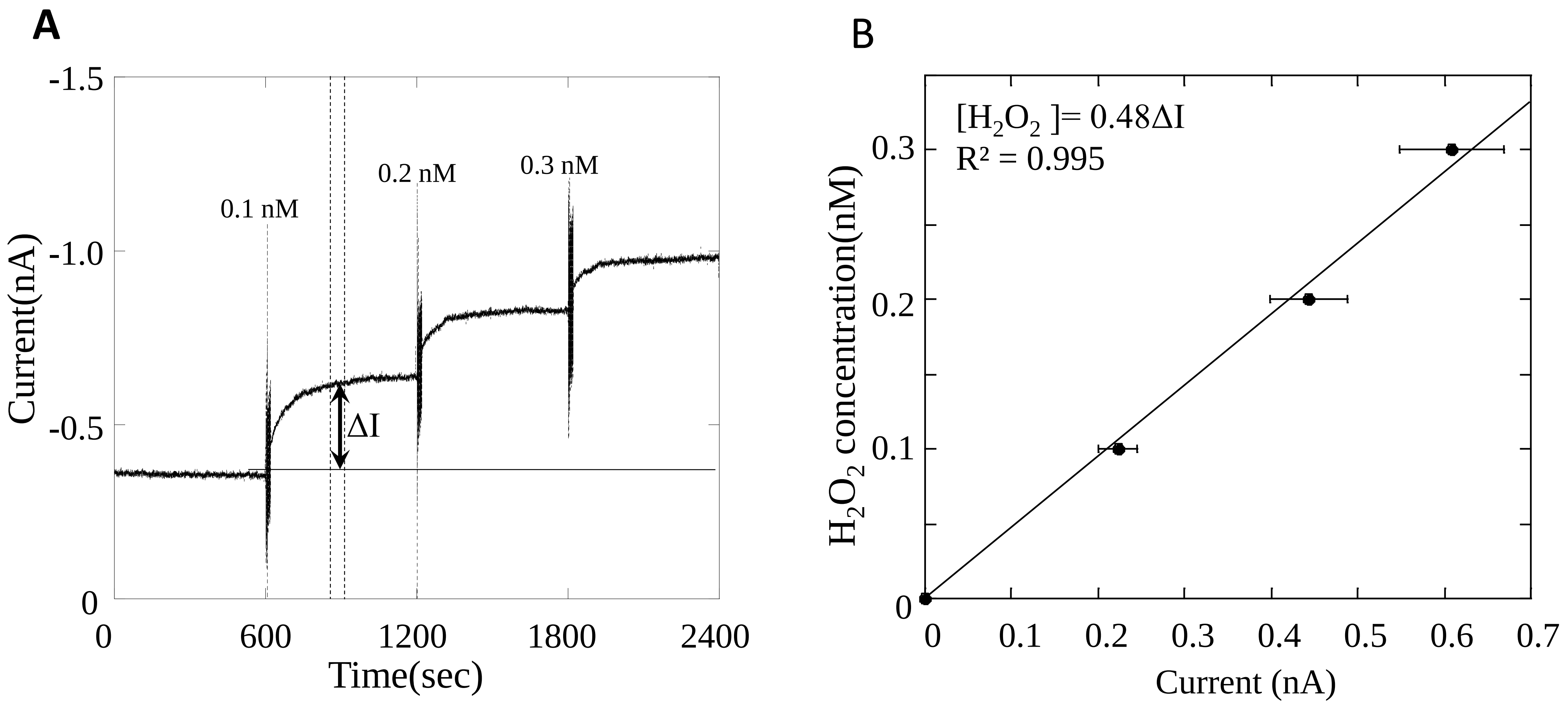

Supplementary data 1: (A), Chronoamperometric response of standard H_2O_2 solution in phosphate buffer in the concentration range of 0.1-0.3 nM. Addition of H_2O_2 solution was done in real-time and subsequent reduction current for H_2O_2 was monitored using Os-HRP modified carbon electrode (φ=1 mm) in non-stirring condition. An Ag/AgCl electrode was used as a reference electrode. (B), Calibration curve was plotted by taking an average of 50 sec of the constant phase (as indicated in A, dotted lines) obtained after the addition of standard H_2O_2 solution.
