# Supplementary material for: Scanning Electrochemical Microscopy Imaging during Respiratory Burst in Human Cell
Source: Front Physiol. 2016 Feb 5;7:25. doi: 10.3389/fphys.2016.00025 (PMC4742556; doi:10.3389/fphys.2016.00025)
Supplement: Supplementary file 2 [file Image2.PDF]

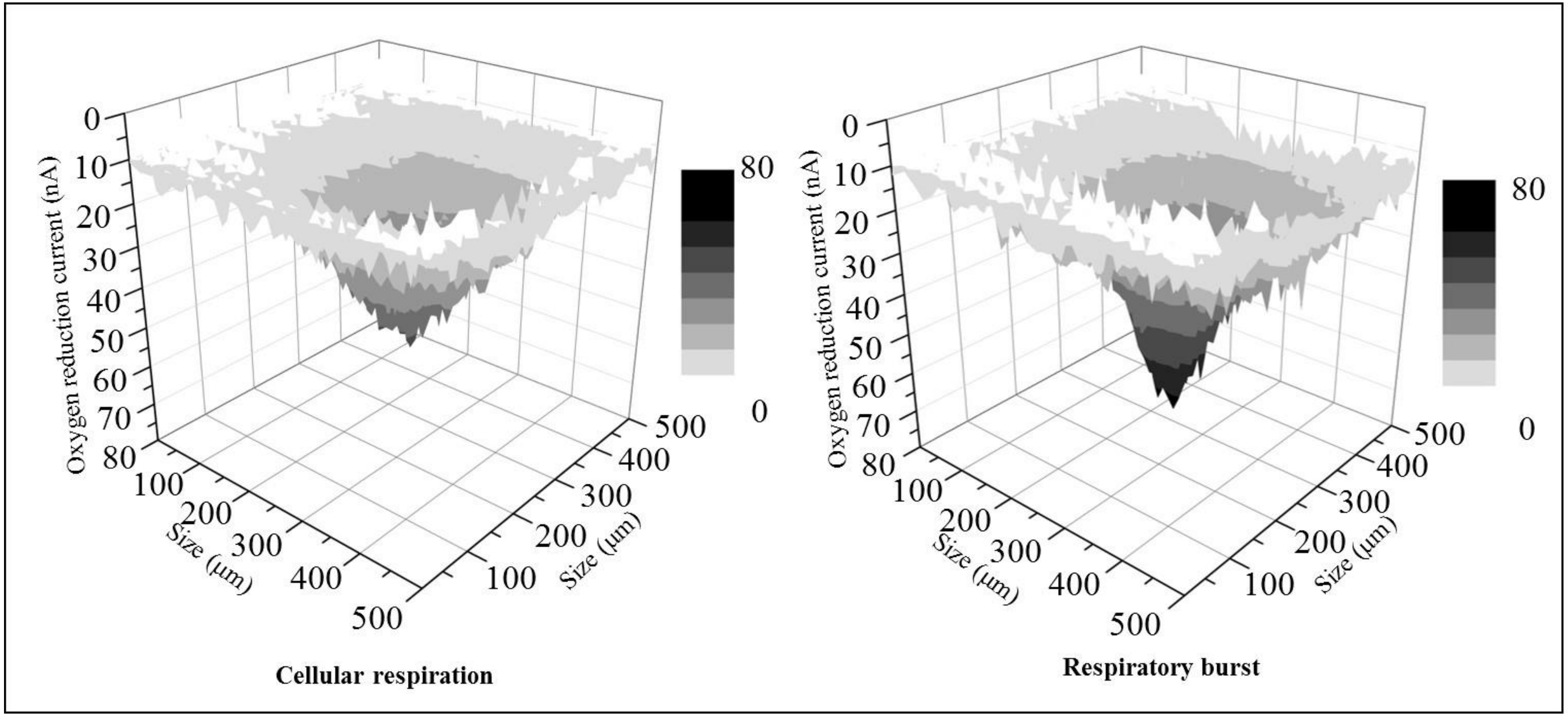

Supplementary data 2: Spatial distribution of changes in oxygen reduction current measured using SECM during cellular respiration (A) and respiratory burst (B). Respiratory burst was induced by exogenous addition of PMA at a final concentrations of 20 nM.
